# Diabetic Kidney Disease: Evidence from Two Selected Cohorts of Patients from Low–Middle and High Income Countries

**DOI:** 10.3390/life15091429

**Published:** 2025-09-11

**Authors:** Maria Mattiotti, Matteo Righini, Daniele Vetrano, Danilo Ribichini, Valentina Vicennati, Valeria Aiello, Ermanno Notaro, Paolo Belardi, Noemi Bazzanini, Katunzi Mutalemwa, Emmanuel Ndile, Rehema Itambu, Uberto Pagotto, Gaetano Azzimonti, Giuseppe Cianciolo, Irene Capelli, Gaetano La Manna

**Affiliations:** 1 Department of Nephrology, Dialysis and Transplantation, San Bortolo Hospital, Via Rodolfi 37, 36100 Vicenza, Italy; maria.mattiotti@aulss8.veneto.it; 2 IRRIV-International Renal Research Institute, 36100 Vicenza, Italy; 3 Doctors with Africa CUAMM, Iringa P.O. Box 11, Tanzania; p.belardi@cuamm.org (P.B.); nbazzanini@ao.pr.it (N.B.); mutalemwa6@gmail.com (K.M.); emmanuelndile@gmail.com (E.N.); rehemajohn.30@gmail.com (R.I.); 4 Nephrology and Dialysis Unit, Santa Maria delle Croci Hospital, AUSL Romagna, 48100 Ravenna, Italy; matteo.righini@auslromagna.it; 5 Department of Medical and Surgical Sciences (DIMEC), Alma Mater Studiorum University of Bologna, 40138 Bologna, Italy; daniele.vetrano@studio.unibo.it (D.V.); valentina.vicennati2@unibo.it (V.V.); ermanno.notaro@studio.unibo.it (E.N.); uberto.pagotto@unibo.it (U.P.); gaetano.lamanna@unibo.it (G.L.M.); 6 Division of Endocrinology and Diabetes Prevention and Care, IRCCS Policlinico Sant’Orsola, Azienda Ospedaliero-Universitaria di Bologna, 40138 Bologna, Italy; danilo.ribichini@aosp.bo.it; 7 Nephrology, Dialysis and Kidney Transplant Unit, IRCCS Azienda Ospedaliero-Universitaria di Bologna, 40138 Bologna, Italy; valeria.aiello6@unibo.it (V.A.); giuseppe.cianciolo@unibo.it (G.C.); 8 Doctors with Africa CUAMM, 35121 Padova, Italy; g.azzimonti@cuamm.org

**Keywords:** diabetic kidney disease, kidney failure, DKD, low-middle income country, vascular complications, kidney protection

## Abstract

**Objectives:** Diabetic kidney disease (DKD) is the leading cause of kidney failure worldwide. Different phenotypes of DKD are emerging, partially attributable to a better glycemic control, partially to concomitant risk factors for kidney disease. Diabetes belongs to Non-Communicable Diseases (NCDs), but poor data about DKD in Low–Middle Income Countries are currently available. In the present paper we compare two cohorts of patients affected by DKD from Tanzania and from Italy. **Study design:** Retrospective observational study conducted by NCDs Clinic of Tosamaganga Regional Referral Hospital (Tanzania) and from the Multidisciplinary Diabetological-Nephrological Clinic of Bologna (Italy). **Methods:** Included patients’ data were analyzed for demographical features, diabetes complications, laboratory findings, and pharmacological therapy at the time of enrollment and after 6-month follow-up. **Results:** Tanzanian patients were younger (56.65 vs. 67.66 years, *p* < 0.001), with a higher prevalence of women (66.9% vs. 25.5%, *p* < 0.001), and showed lower level of BMI (26.39 vs. 30.18 kg/m^2^, *p* < 0.001). Worsened glycemic control could be observed in the Tanzanian cohort (HbA1c 83.71 vs. 56.92 mmol/mol, *p* < 0.001) and higher eGFR (70.13 ± 31.93 vs. 52.31 ± 23.37 mL/min, *p* < 0.001). A sharp reduction in albuminuria was observed in both cohorts with an increase in nephroprotective drugs and better glycemic control. **Conclusions:** Two phenotypes of diabetic patients have emerged from comparison between two cohorts. Tanzanian patients are mostly female, younger, and with a normal BMI, whereas Italian patients are mainly male, older, and affected by metabolic syndrome and vascular complications. Therapy implementation is associated with a delayed decline of eGFR and downgrading of albuminuria at 6-month follow-up.

## 1. Introduction

According to the last WHO Global Report, the number of patients affected by diabetes mellitus (DM) has quadruplicated since 1980, reaching 422 million of patients in 2019, and it will rise to 693 million in the next two decades [1]. Among diabetic patients, 90–95% are affected by Type 2 Diabetes Mellitus (T2DM). Increased morbidity and mortality are mainly attributable to microvascular and macrovascular complications [2]. Unlike other diabetes complications, the prevalence of diabetic kidney disease (DKD) has failed to decline over past decades and it still represents worldwide the leading cause of kidney failure [3]. Hyperglycemia plays a pivotal role in the pathogenesis of DKD. Persistent high serum glucose level induces metabolic changes and triggers a systemic inflammatory response; these two conditions in turn exacerbate progression of glomerular damage and decline of glomerular filtration rate (GFR). The relative contribution of each factor in renal injury cannot be a priori established [4].

Moreover, the classical paradigm of “albuminuric diabetic nephropathy” [5] has been replaced by a wider spectrum of nephropathies, which includes at least two other patterns of disease: “nonalbuminuric renal impairment” and “progressive renal decline” diabetic kidney disease [2]. Those phenotypes may have been unmasked by the regression of albuminuria obtained by improved glycemic control, mostly attributable to pharmacological treatment. Otherwise, a high proportion of diabetic patients with chronic kidney disease (CKD) show underlying kidney pathologies other than DKD when systematically subjected to kidney biopsy [6]. Furthermore, between diabetic population, other comorbidities, such as atherosclerotic cardiovascular disease, heart failure, and obesity [6,7] enhance renal injury and increase mortality rate [8].

In addition, DM and CKD belong to Non-Communicable Diseases (NCDs), chronic non-infectious diseases with a high rate of morbidity and early mortality rate worldwide. The highest burden of death for these conditions affects Low–Middle Income Counties (LMIC) [9,10,11]. NCDs include group of chronic conditions, such as cardiovascular diseases, cancer, chronic respiratory diseases, diabetes, and CKD, accounting for 71% of deaths worldwide [9,10]. A large proportion of these deaths occur in LMICs, where about 700 million people experience extreme levels of poverty [9,12]. Tanzania is an LMIC, and in the last 25 years, the incidence of NCDs has doubled. The lack of prevention, the pathogenetic association with infectious diseases, and the limited availability of facilities in rural regions, may play a pivotal role more than lifestyle risk factors [13].

Since 2016, Doctors with Africa CUAMM (CUAMM), in partnership with local authorities, started a dedicated clinical program for NCD prevention and treatment at Tosamaganga Regional Referral Hospital (Tosamaganga Hospital). In order to promote long-term follow-up as cornerstone for a good prevention of chronic diseases, based on the HIV-care model [14], which is characterized by a strong network with peripheral Health Centers, CUAMM has started an integrated management system of NCDs at Tosamaganga Hospital and its reference health district, namely Iringa District Council (Iringa DC).

Additionally, poor data are nowadays available about DKD prevalence in LMICs [15]: besides under diagnosis [16], limited efforts are aimed at DKD prevention and management, compared to AKI [17,18,19,20,21,22]. The most available data about prevalence of DKD in Tanzania are single-center studies [23,24]. In Team 2020, the Diabetology Unit and Nephrology Unit of University Hospital of Bologna (Italy) started a pilot experience of a multidisciplinary outpatient service to provide an integrated approach to DKD.

The aim of this study is to present and analyze the preliminary results of the two cohorts of diabetic patients with CKD with a focus on the epidemiological, social, demographical, clinical and diagnostic–therapeutic features of each population.

## 2. Methods

This is an observational, retrospective, multicenter study involving IRCCS St. Orsola University Hospital in Bologna, Italy, and Tosamaganga Hospital, Iringa District Council (Iringa DC), Iringa Region, Tanzania.

The Italian cohort included all the patients referred to multidisciplinary outpatient service from 1 February 2020 to 30 April 2022. The inclusion criteria were age over 18 years, with a diagnosis of diabetes mellitus Type 1 (T1DM) or T2DM, signs of renal impairment, or an established CKD with evidence of worsening of glycemic control, and at least one evaluation by the Integrated Diabetological and Nephrological Multidisciplinary Outpatient Service. As a sign of renal damage, at least one of the following conditions should be present: increased serum creatinine level (sCr) and/or lower estimated GFR (eGFR) from basal level, new onset urine test abnormalities, worsening of microalbuminuria, increased urine albumin–creatinine ratio (UACR), new onset or proteinuria worsening or presence of microhematuria, or evidence of kidney morphological abnormalities ultrasound proven.

The Tanzanian cohort included all diabetic patients referred to outpatient service for treatment and prevention of NCDs (NCDs Clinic) service from 1 March 2019 to 31 July 2022. The inclusion criteria were age over 18 years, diagnosis of T1DM or T2DM, at least one evaluation by a sign of renal impairment, and at least one evaluation by the NCDs Clinic. As a sign of renal damage, at least one of the following features should be present: eGFR < 60 mL/min and/or urine test abnormalities (presence of albuminuria at urinary stick).

The study procedures were in accordance with the Helsinki Declaration. In Italy the study protocol and consent form were approved by the Ethics Committee of St. Orsola University Hospital (ID approval: 1008-2021). All patients were informed about the experimental protocol and about the objectives of the study before providing informed consent.

In Tanzania, all data were retrieved from hospital records by clinical staff and were collected in an anonymized dataset. The study was approved by the Review Board of Tosamaganga Hospital (protocol number DOIRA/TRRH/VOL.56/122), which waived the need for written informed consent given the retrospective nature of the study and the use of anonymized data from hospital records.

### 2.1. Integrated Multidisciplinary Outpatient Service

#### 2.1.1. Physician Team

The physician team involved in the integrated multidisciplinary outpatient service was composed by Nephrologists and Diabetologists operating within their respective units (Division of Nephrology, Dialysis and Transplant and Division of Endocrinology and Diabetes Prevention and Care). Most of them had previous expertise on integrated outpatient service, involving different clinical areas. All physicians were involved in the process of conception of this project: choice of patient selection criteria, data collection, clinical and laboratory evaluation criteria, and therapeutic rational of the integrated multidisciplinary outpatient service.

#### 2.1.2. Visit Protocol

All visits were performed by a Diabetologist and a Nephrologist simultaneously present at the time of the patient evaluation.

During the first evaluation (Visit 0) the detailed medical history was collected. Each patient was screened for the main diabetes complications (retinopathy, neuropathy, diabetic foot, Major Adverse Cardiac Event (MACE), heart failure, peripheral artery disease (PAD), stroke, Transient Ischemic Attack (TIA), steatosis), and for the main risk factors of renal injury (smoke, hypertension, dyslipidemia). They were questioned about ongoing pharmacological treatment (antidiabetic drugs, anti-hypertensive treatment, lipid-lowering agents). Each patient underwent physical examination, including blood pressure (BP) measurement, weight, height, body mass index (BMI) calculation, and waist circumference measurement. The following blood results were also collected: serum fasting glucose, glycosylated hemoglobin (HbA1c), sCr, eGFR, urine test, microalbuminuria (mg/dL), UACR (mg/g), proteinuria (mg/24 h), lipid profile assessment, hemoglobin, and electrolytes. At the end of the visit, pharmacological therapy was optimized, if needed, for glycemic control; for renal disease and for cardiovascular risk factors, further investigation could be prescribed, including a dietologist or other specialistic consultations.

The follow-up evaluation (Visit 1) was performed 6 months after V0, and it could be either multidisciplinary or, as usually organized in standard care system, a distinct diabetologic and nephrologic visit. In both cases, new onset of cardiovascular or cerebrovascular events and diabetes complications were recorded; pharmacological reconciliation was performed; physical examination, BP measurement, weight and waist circumference measurement and BMI calculation were obtained; and updated laboratory blood tests were collected.

### 2.2. Integrated NCDs Clinic

#### 2.2.1. Clinical Team

The clinical team involved in NCDs Clinic included a Physician, with specialization in Internal Medicine or equivalent to it, two nurses with a specific education on NCDs, and a trained Nutritionist for dietary and lifestyle counseling.

#### 2.2.2. Visit Protocol

All patients with evidence of hypertension or impaired glycemic control could be referred to this clinic. During the first evaluation (Visit 0) the most relevant events of their medical history were collected. Each patient was screened for the main diabetes complications (retinopathy, neuropathy, diabetic foot, MACE, heart failure, PAD, stroke, TIA, CKD) and for the main risk factors of renal injury (smoke, alcohol abuse, sedentary lifestyle). They were questioned about ongoing pharmacological treatment (antidiabetic drugs, anti-hypertensive treatment, lipid-lowering agents). Each patient underwent physical examination, including BP measurement, body weight, height, BMI calculation.

The following blood results were also collected: serum fasting glucose, HbA1c, sCr, eGFR, urine stick test (negative: <15 mg/dL; +1: 15–100 mg/dL; +2: 100–300 mg/dL; +3: 300–1000 mg/dL; +4: >1000 mg/dL), lipid profile assessment, hemoglobin. At the end of the visit pharmacological therapy was optimized, if needed, for glycemic control, hypertension, renal and cardiovascular risk factors control, and a detailed lifestyle and nutritional counseling were offered. The follow-up evaluations (Visit 1) were performed every 6 months at Tosamaganga Hospital, while monthly intermediate controls could be accomplished by peripheral Health Centers. Along these visits, new onset of cardiovascular or cerebrovascular events and diabetes complications were recorded; pharmacological reconciliation was performed; physical examination, BP measurement, weight and BMI calculation were obtained; and updated laboratory blood tests were collected.

### 2.3. Variables Analyzed

The Italian and Tanzanian cohorts were analyzed and compared at Visit 0 (V0) and Visit 1 (V1) at 6 months, if available, for the following variables: anthropometric features (BP measurement, weight, height, BMI); laboratory tests (serum fasting glucose, HbA1c, sCr, eGFR, urine stick test); diabetes complications (retinopathy, neuropathy, diabetic foot, MACE, PAD, stroke, TIA); ongoing pharmacological treatment, including antidiabetic drugs:, sulfonylureas and insulin, and nephroprotective therapy: Renin-Angiotensin-Aldosteron System inhibitors (RAASi), and Sodium Glucose Transporter 2 inhibitors (SGLT2i) and Glucagon-like peptide-1 receptor agonists (GLP1-RA), and statin. Only metformin and solphanilureas as oral hypoglycemics agents have been compared because those available in Tanzania. Albuminuria over time was also compared between two populations. They were stratified into three groups of albuminuria, namely normoalbuminuric, microalbuminuric, and macroalbuminuric. In the Italian population, normoalbuminuric corresponded with class A1 according to the KDIGO classification (UACR < 30 mg/g), microalbuminuric with class A2 (30–300 mg/g), and macroalbuminuric with class A3 (>300 mg/g). Tanzanian population was stratified with urine dipstick findings, with normoalbuminuric corresponding to a negative dipstick result, microalbuminuric with 1+ albuminuria, and macroalbuminuric with 2+ or more albuminuria.

### 2.4. Statistical Analysis

Descriptive statistics were employed to compare the Italian and Tanzanian cohorts both initially and during follow-up. Categorical variables were represented as percentages, while continuous variables were presented as either means with standard deviations for parametric variables, or medians with interquartile ranges (IQR) for nonparametric variables. To assess variations between the groups, the Kruskal–Wallis test and unpaired *t*-test were utilized for both skewed and symmetric variables. Additionally, the Chi-squared test was applied to analyze differences in percentages between the two groups.

A *p*-value of <0.05 was considered statistically significant.

Statistical analysis was performed using R version 4.0.3 (R Foundation for Statistical Computing, Vienna, Austria).

## 3. Results

Between March 2019 and July 2022, 139 Tanzanian patients out of 417 diabetic patients referring to NCDs showed eGFR < 60 mL/min or albuminuria at urinary stick. They have been compared to 235 Italian patients referring to integrated multidisciplinary outpatient service between 1 February 2020 and 30 April 2022.

### 3.1. Demographical Features and Complications

Main results are summarized on Table 1.

Tanzanian patients were significantly younger with a mean age of 56.65 ± 13.93 years against 67.66 ± 12.21 years (*p* < 0.001). Moreover, the Tanzanian cohort was female prevalent (66.9%) while the Italian one was male prevalent (74.5%), and this difference was statically significant (*p* < 0.001).

In terms of comorbidities, the Tanzanian population exhibited a significantly higher prevalence of retinopathy (38.8% vs. 26.5%, *p* = 0.015), whereas the Italian cohort had a higher incidence of MACE (30.3% vs. 0.7%, *p* < 0.001). However, there were no significant differences observed in the rates of diabetic foot or heart failure between the two groups. Notably, the Italian cohort had a higher prevalence of smoking, with a greater proportion of former smokers (44% vs. 0%) and current smokers (14.1% vs. 6.5%, *p* < 0.001).

From a metabolic perspective, Tanzanian patients presented significantly lower BMI at the initial evaluation compared to their counterparts (26.39 vs. 30.18, *p* < 0.001). However, they exhibited significantly higher HbA1c levels (83.71 vs. 56.92 mmol/mol, *p* < 0.001). Additionally, they showed significantly lower levels of total cholesterol (159.1 vs. 168.2 mg/dL, *p* = 0.027).

### 3.2. HbA1c over Time

As mentioned above, the Tanzanian cohort exhibited significantly higher levels of HbA1c at baseline compared with Italian cohort (83.71 vs. 56.92 mmol/mol, *p* < 0.001). At V1, however, there was a marked reduction in HbA1c levels in the Tanzanian cohort, while the reduction was lower in Italian cohort (59.07 vs. 55.77 mmol/mol, *p* = 530).

### 3.3. Renal Function over Time

As depicted in Table 2, V0 Italian patients exhibit more severe kidney impairment, characterized by significantly higher mean creatinine values and lower eGFR compared to Tanzanian patients (1.49 vs. 1.38 mg/dL, *p* < 0.001 and 52.31 vs. 70.12 mL/min/1.73m^2^, respectively). Similarly, at V1, significantly higher values of serum creatinine (1.52 vs. 1.20 mg/dL, *p* < 0.001) and lower values of eGFR (49.9 vs. 79.3 mL/min/1.73 m^2^, *p* < 0.001) were observed.

### 3.4. Albuminuria over Time

**Figure 1:**
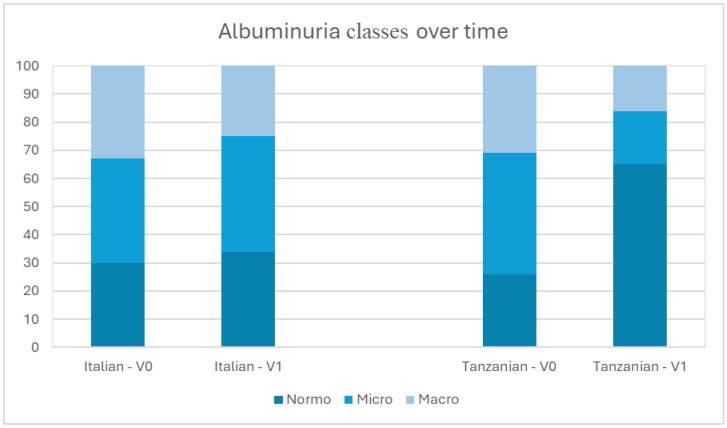
Albuminuria classes over time.

At V0, the prevalence of albuminuria in the Italian and Tanzanian populations was as follows: 29.7% vs. 26.5% for normoalbuminuric, 37.4% vs. 42.6% for microalbuminuric, and 33.0% vs. 30.9% for macroalbuminuric, respectively. There was no statistical significance between the two. At V1, the prevalence between the Italian and Tanzanian cohorts was as follows: normoalbuminuric (34 vs. 65%), microalbuminuric (41% vs. 19%), and macroalbuminuric (25% vs. 16%) (Figure 1). The differences were statistically significant (*p* = 0.028).

### 3.5. RAASi over Time

The prevalence of Angiotensin Converting Enzyme inhibitor (ACEi) and Angiotensin Receptor Blocker (ARB) therapy was compared between the two populations. At V0, 40% of Italian patients were already on ACEi therapy, while at V1, the prevalence increased to 47%. For the Tanzanian population, only 20% of patients were on ACEi therapy at baseline, whereas at the follow-up, this proportion increased to 41%. Similarly, the percentage of patients on ARB therapy increased from 37.9% at baseline to 43% at the follow-up assessment in the Italian cohort, while in the Tanzanian population, it remained almost the same, with 35% at V0 and 36% at V1.

**Figure 2:**
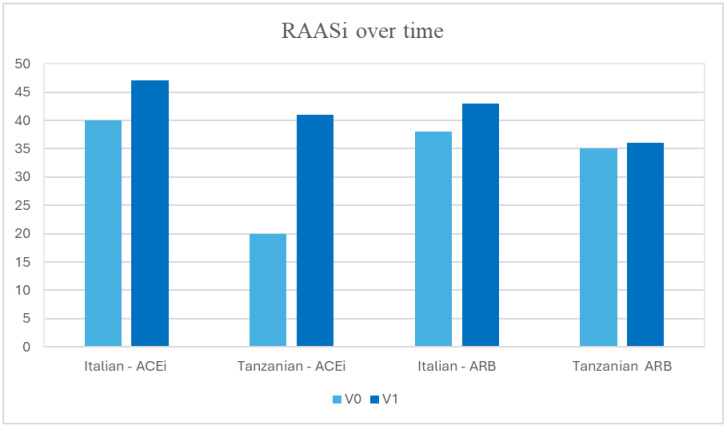
RAASi over time.

In summary, the prevalence of RAASi therapy improved from 80% to 100% in the Italian population and from 55% to 77% in the Tanzanian population (Figure 2).

### 3.6. SGLT2i over Time

Limited to Italian cohort, prescription of SGLT2i has been analyzed. The major increase in their assumption has been documented between V0 and V1: from 18% at V0 to 48% at V1.

## 4. Discussion

In the present study we showed a comparison between two selected cohorts of diabetic patients referring to an Integrated Multidisciplinary Outpatient in Italy and from Integrated Clinic of NCDs in Tanzania, by analyzing epidemiological, social, demographical, clinical, and diagnostic–therapeutic features of each population.

To the best of our knowledge there are no studies which compare features of patients affected by DKD in LMICs and a High Income Countries.

Expanding knowledge about DKD, its high prevalence, and the burden of chronic comorbidities in diabetic patients suggested an integrated approach to DKD involving different specialists for a best management of this challenging disease. It has been shown that this approach is associated with a delayed decline of kidney function, improved metabolic control [24,25,26,27,28], deferred dialysis initiation [29], and outpatient cost saving [30]. More recently a new Australian co-designed, person-centered integrated model of care has proven to improve all-cause mortality, kidney function, glycemic control, self-care, and health-related quality of life on patients with diabetes and CKD [31,32].

The two populations are only partially comparable because it is a retrospective study of patients referring to services with different rational and different care models, based for the Bologna model on un integrated multidisciplinary assessment by Nephrologists and Diabetologists, and for the Tosamaganga model with periodic internal medicine evaluations. A comprehensive comparison and an advanced analysis of the difference go beyond the scope of this paper. However, we believed that it could help to improve data collections and research projects in this field.

The mean age of patients in Bologna is higher than that observed in Tosamaganga. This result is consistent firstly with lower mean life expectancy at birth of Tanzanian population (66 years) if compared with the Italian population (82 years), and for different median ages of diabetes development for both genetic and lifestyle causes. A significant prevalence of women between Tanzanian patients and a higher percentage of men between Italian people could be observed: this difference may not be explained by a different sex prevalence of disease between two cohorts, but it could reflect the so-called “health-seeking behavior” [33]. This expression refers to diffuse behavior of Sub-Saharan women to be compliant to follow up programs, always starting with pregnancy care. The average BMI of Italian diabetics is higher than 30 kg/m^2^, while Tanzanian patients show a median BMI lower than 26 kg/m^2^. The percentage of patients affected by T2DM and T1DM were comparable in two populations. A lot of evidence is available about a form of diabetes secondary to malnutrition [34] that could justify the lower BMI of Tanzanian diabetic patients. The putative mechanism may be a reduced dietary intake of mother during pregnancy or during early childhood that could develop a reduced glucose tolerance, through epigenetic modifications influencing the development and differentiation of pancreatic beta cells. On enrollment, BP measurements register higher mean values for Tanzanian patients, if compared to the Italian, but it could be a bias of selection: patients from Bologna were already on diabetological or nephrological follow-up and may have already reached BP target, otherwise patients referred to the NCDs Clinic always had their first evidence of hypertension on enrollment. The same observation could explain the difference average HbA1c between two populations: significantly higher level have been found in Tosamaganga patients at baseline, always first diagnosed for diabetes at enrollment, while all patients from Bologna where all on chronic glucose-lowering treatment and showed a more adequate glycemic control.

Diabetic patients from Bologna showed lower mean eGFR both at enrollment and on follow-up. The most represented classes of CKD were 3a and 3b, while for diabetic patients from Tosamaganga, CKD stage 1 and 2 prevailed. Analogous distributions between CKD Class are documented after 6 months of follow-up in both cohorts. The more advanced grade of CKD in Italian patients is due to the rationale of Integrated Multidisciplinary Outpatient Service, whose enrolment was justified by an impaired renal function in diabetic patients not completely explained by a worsened glycemic control and a patient’s phenotype characterized by a worse cardiovascular profile. Otherwise, patients of NCDs Clinic are selected, younger diabetic patients with impaired renal function at enrollment, uncontrolled glycemic level, and untreated hypertension.

Regarding albuminuria, a substantial stability of normoalbuminuric patients could be observed between Italian patients; during follow-up an increased percentage of microalbuminuric and a decreased percentage of macroalbuminuric are highlighted, which suggests a class downgrading within the cohort. In the Tanzanian cohort a decline of macroalbuminuric patients during the first 6 months of follow-up has been observed. This could be secondary to an improved glycemic control obtained through adequate diet and pharmacological therapy; the group of microalbuminuric patients could moreover include patients with an incipient nephropathy and with a negative urinary stick on subsequent follow-up. Albuminuria detection for patients in Tosamaganga has been performed with a urinary stick, which gives only a semiquantitative result with a moderate sensitivity (62%, 36%, and 78%) and high specificity (88%, 88%, and 98%) [35,36]. Substantial stability of eGFR and improvement of albuminuria at one year follow-up of both cohorts suggest an advantage on taking charge of diabetic patients for an improved control of albuminuria and slow progression of CKD.

These results are coherent to what has been already stated in studies performed in North Africa [37,38] and in Sub Saharan Africa [22,23,39], where DKD prevalence and features are different between Caucasian and Afro-American races. Socioeconomic factors, including different lifestyle and risk factors exposure, and different sanitary systems, different access to screening and facilities, and timing of diagnosis, are the main potential causes. A genetic contribution cannot be excluded to explain those different phenotypes. Evidence of increased risk of end-stage renal disease (ESRD) in diabetic patients of Afro-American race living in HICs have been published [40]. An association between DKD development and polygenic inheritance has been observed, and small familiar clusters association between specific genetic mutation and specific renal involvement (increased UACR, reduced eGFR, microalbuminuria) have been observed [41]. Moreover, an association between development of ESRD in the Afro-American race and mutation of APOL1 e GNG7 has been extensively described [42]. Finally, some other mechanisms of diabetes, like those that are malnutrition related, cannot be excluded.

It is also necessary to remember how the selection of patients with kidney damage as carried out may have included patients with non-diabetic or mixed etiology of the damage.

Diabetes microvascular and macrovascular complications usually show different prevalences between LMICs and HICs; in particular, diabetic foot onset can be reduced with preventive measures and multispecialist care [43]. Interestingly, no statistical difference in diabetic foot prevalence has been observed comparing our cohorts [44].

In the context of nephroprotective strategies, the utilization of RAASi and SGLT2i has been examined. At baseline (V0), the majority of patients in the Italian cohort received ACEi or ARB, whereas this percentage was lower in the Tanzanian cohort. At follow-up, in both cohorts, there was an optimization of RAASi therapy, with the Italian cohort achieving full coverage across the population, while the Tanzanian cohort experienced a significant improvement in their assumption. The use of SGLT2i was studied only in the Italian population and a higher percentage of employment was observed. Unfortunately, SGLT2i were not available in rural district of Iringa Region. The lack of use of this therapy in the Tosamaganga cohort may have an impact on the results in terms of outcomes and therefore on the comparison between the cohorts. Other studies must be performed to confirm the data in the Tanzanian population. In the Italian cohort a contribution in renal preservation could be supposed also by GLP1-RA action [45].

The substantial increase in the utilization of nephroprotective drugs, combined with the optimization of HbA1c control, could potentially explain the significant reduction in albuminuria observed in the Tanzanian cohort, and the documented improved kidney function. However, the Italian population also benefited from therapeutic optimization, showing an improvement in albuminuria classes along with stable renal function.

### Limitations

The main limitation of this work is related to the retrospective nature of the study itself. The different demographical features between the two cohorts are mainly attributable to different geographical economic contexts. Other limitations are related to different available laboratory methods of analysis in two contexts, namely limited accuracy and the sensitivity of the diagnostic tool for Tosamaganga compared to Italian tests. Furthermore, a reliable comparison for albuminuria was not possible because two different parameters have been used for albuminuria detection.

## 5. Conclusions

Two phenotypes of diabetic patients with DKD have been highlighted from comparison between one LMIC and one HIC: Italian patients are older and mainly affected by cardiovascular comorbidities, including metabolic syndrome and vascular complications, while the prevalent phenotype of Tanzanian patients affected by DKD is characterized by female sex, young age, and normal BMI.

Stability of eGFR and downgrading of albuminuria classes at one year follow-up are reached in both cohorts with a certain contribution of optimization of nephroprotective strategy, namely the introduction of RAASi in Tanzania and SGLT2i in Italy. Adherence to follow-up and compliance with therapy seem to play a central in improving the outcomes for both populations.

Further studies are necessary for a better understanding of the different physiopathology and clinical features between LMICs and HICs. Furthermore, prospective trials or randomized control trials may be useful for a deeper knowledge about prevalence and clinical features of this disease especially in LMICs. From their results an implementation of sustainable programs in African healthcare systems could be reached. Expanding knowledge about DKD could help to optimize therapy and delay disease progression for these patients.

## Figures and Tables

**Table 1:** Baseline features.

	Tosamaganga (*N* = 139)	Bologna (*N* = 235)	Total (*N* = 374)	*p*-Value
Sex, *n* (%)				<0.001
F	93 (66.9%)	60 (25.5%)	153 (40.9%)	
M	46 (33.1%)	175 (74.5%)	221 (59.1%)	
Age, years	56.65 (13.93)	67.66 (12.21)	63.56 (13.92)	<0.001
Smoke, *n* (%)				<0.001
- Current	9 (6.5%)	33 (14.1%)	42 (11.3%)	
- Former	0 (0.0%)	103 (44.0%)	103 (27.6%)	
Retinopathy, *n* (%)	54 (38.8%)	61 (26.5%)	115 (31.2%)	0.015
Diabetic foot, *n* (%)	7 (5.0%)	20 (8.7%)	27 (7.3%)	0.296
HF, *n* (%)	10 (7.2%)	13 (8.8%)	23 (8.0%)	0.668
MACE, *n* (%)	1 (0.7%)	70 (30.3%)	71 (19.2%)	<0.001
Stroke, *n* (%)	10 (7.2%)	18 (12.0%)	28 (9.7%)	
BMI, kg/m^2^ (SD)	26.39 (5.58)	30.18 (5.80)	28.75 (6.00)	<0.001
Hba1c, mmol/mol (SD)	83.71 (32.03)	56.92 (13.79)	65.96 (25.12)	<0.001
Total Cholesterol, mg/dL (SD)	159.18 (57.53)	168.12 (44.91)	164.58 (50.40)	0.028
sCr, mg/dL (SD)	1.38 (0.98)	1.49 (0.52)	1.45 (0.73)	<0.001
eGFR, mL/min/1.73m^2^ (SD)	70.13 (31.93)	52.31 (23.37)	58.97 (28.20)	<0.001
Albuminuria class, *n* (%)				0.617
- Normo	36 (26.5%)	54 (29.7%)	90 (28.3%)	
- Micro	58 (42.6%)	68 (37.4%)	126 (39.6%)	
- Macro	42 (30.9%)	60 (33.0%)	102 (32.1%)	
ACEi, *n* (%)	16 (20.0%)	94 (40.0%)	110 (34.9%)	0.001
ARB, *n* (%)	28 (35.0%)	89 (37.9%)	117 (37.1%)	0.689
CCB, *n* (%)	36 (45.0%)	69 (42.6%)	105 (43.4%)	0.783
Diuretics, *n* (%)	24 (30%)	69 (43%)	93 (38.5%)	0.045
Metoformin, *n* (%)	112 (80.6%)	114 (48.5%)	226 (60.4%)	<0.001
Sulfonylureas, *n* (%)	75 (54.0%)	31 (13.2%)	106 (28.3%)	<0.001
Insulin, *n* (%)	9 (6.5%)	102 (43.4%)	111 (29.7%)	<0.001
SGLT2i, *n* (%)	-	42 (18.0%)	42 (11.2%)	**-**
GLP1-RA, *n* (%)	-	30 (12.7%)	30 (8.0%)	-

M, male; F, female; HF, Heart Failure; MACE, Major Adverse Cardiac Event; BMI, Body Mass Index; SD, standard deviation; HbA1c, Hemoglobin glycated; sCr, serum creatinine; eGFR, estimated glomerular filtration rate; ACE, Angiotensin Converting Enzyme inhibitor; ARB, angiotensin receptor blockers; CCB, calcium channel blockers; SGLT2i, Sodium Glucose Transporter 2 inhibitors; GLP1-RA, Glucagon-like peptide-1 receptor agonists.

**Table 2:** Follow-up features.

	Tosamaganga (*N* = 139)	Bologna (*N* = 235)	Total (*N* = 374)	*p*-Value
BMI kg/m^2^ (SD)	27.35 (5.88)	29.64 (5.00)	28.86 (5.41)	0.015
Hba1c, mmol/mol (SD)	59.07 (28.80)	55.77 (12.18)	56.65 (18.12)	0.53’
Total Cholesterol, mg/dL (SD)	155.71 (52.65)	170.00 (47.09)	166.73 (48.61)	0.061
sCr, mg/dL (SD)	1.20 (0.92)	1.52 (0.55)	1.43 (0.69)	<0.001
eGFR, mL/min/1.73m^2^ (SD)	79.38 (34.17)	49.90 (21.37)	58.80 (29.17)	<0.001
Albuminuria class, *n* (%)				0.617
- Normo	65.0%	34.0%	28.3%	
- Micro	19.0%	41.0%	39.6%	
- Macro	16.0%	25.0%	32.1%	
ACEi, (%)	41.0%	47.0%	45.0%	0.001
ARB, (%)	36.0%	43.0%	41.9%	0.689
SLT2i, *n* (%)	-	112 (48%)	112 (30%)	-

BMI, Body Mass Index; HbA1c, Hemoglobin glycated; sCr, serum creatinine; eGFR, estimated glomerular filtration rate; ACE, Angiotensin Converting Enzyme inhibitor; ARB, angiotensin receptor blockers; SGLT2i, Sodium Glucose Transporter 2 inhibitors.

## Data Availability

The data presented in this study are available on request from the corresponding author. The datasets generated and/or analyzed during the current study are not publicly available due to privacy concerns but are available from the corresponding author on reasonable request.
